# A Dominant EV71-Specific CD4+ T Cell Epitope Is Highly Conserved among Human Enteroviruses

**DOI:** 10.1371/journal.pone.0051957

**Published:** 2012-12-14

**Authors:** Ruicheng Wei, Chunfu Yang, Mei Zeng, Frances Terry, Kai Zhu, Chunhui Yang, Ralf Altmeyer, William Martin, Anne S. De Groot, Qibin Leng

**Affiliations:** 1 Key Laboratory of Molecular Virology and Immunology, Institut Pasteur of Shanghai, Shanghai Institutes for Biological Sciences, Chinese Academy of Sciences, Shanghai, People’s Republic of China; 2 EpiVax, Inc., Providence, Rhode Island, United States of America; 3 Department of Infectious Diseases, Children's Hospital of Fudan University, Shanghai, People’s Republic of China; 4 Institute for Immunology and Informatics, University of Rhode Island, Providence, Rhode Island, United States of America; University of Rochester, United States of America

## Abstract

CD4+ T cell-mediated immunity plays a central role in determining the immunopathogenesis of viral infections. However, the role of CD4+ T cells in EV71 infection, which causes hand, foot and mouth disease (HFMD), has yet to be elucidated. We applied a sophisticated method to identify promiscuous CD4+ T cell epitopes contained within the sequence of the EV71 polyprotein. Fifteen epitopes were identified, and three of them are dominant ones. The most dominant epitope is highly conserved among enterovirus species, including HFMD-related coxsackieviruses, HFMD-unrelated echoviruses and polioviruses. Furthermore, the CD4+ T cells specific to the epitope indeed cross-reacted with the homolog of poliovirus 3 Sabin. Our findings imply that CD4+ T cell responses to poliovirus following vaccination, or to other enteroviruses to which individuals may be exposed in early childhood, may have a modulating effect on subsequent CD4+ T cell response to EV71 infection or vaccine.

## Introduction

HFMD, caused by enteroviruses, is a common illness in children who are less than 5 years old. HFMD has currently emerged as a major infectious disease in China, affecting about one million children annually [1]–[3]. Currently, there is neither a prophylactic vaccine nor antiviral therapy to treat the disease. Understanding the immunopathogenesis of HFMD and the potential cross-reactive immunity to other viral infections and vaccines may assist in developing a novel therapy or effective vaccine against HFMD.

The major etiological agents of HFMD are enterovirus 71 (EV71), coxsackievirus (CV) A16 [4], [5], and other enteroviruses including CV serotypes A2, A4, A6, A9, A10, B1, B3 and B5, also cause mild HFMD [6]–[11]. EV71 is the most pathogenic virus of these causative agents, causing severe HFMD with complications including brainstem encephalitis, severe pulmonary edema, and significant mortality [12], [13].

Several studies suggest that the cellular immune response plays an important role in controlling EV71 infection. First, the level of antibody against EV71 does not correlate with disease severity [2], [14]. Peripheral blood mononuclear cells (PBMCs) from severe HFMD patients tend to have lower proliferation in response to inactivated EV71 virus [14], suggesting an inverse correlation between antigen-specific T cell response and disease severity. Secondly, a case-control study has revealed that selected MHC class I-restricted CD8+ and class II-restricted CD4+ T cell responses are involved in susceptibility to EV71 infection [15]. Furthermore, mice that lack either CD4+ or CD8+ T cells develop severe disease upon infection with mouse-adaptive EV71 [16].

CD4+ T cells are central players in the adaptive immune response, providing helper signaling for B cells to produce antibody and CD8+ T cells to become cytotoxic killer cells, and also by modulating the responses of recruited innate immune cells [17], [18]. Understanding how CD4+ T cells respond to EV71 infection not only sheds light on the pathogenic process but also on effective vaccine development. Previously, three epitopes within EV71 VP1 protein were predicted with an online algorithm and validated for their abilities to induce proliferation of human CD4+ T cells [19]. This contrasts with an earlier study that had suggested that cross-reactive T cell epitopes exist for enteroviruses, and that the cross-reactive CD4+ T cell epitopes are located in VP2 and VP3 proteins rather than VP1 proteins [20]. Epitope mapping for EV71 should also take into consideration related enteroviruses such as Polio virus, since poliovirus vaccination is widespread and most children are exposed to additional enteroviruses during childhood. Thus, in this study, we considered how immunization or exposure to other enteroviruses may affect CD4+ T cell response to EV71 infection of HFMD patients and the efficacy of EV71 vaccines in clinical trials, and examined whether cross-conservation contributes to epitope dominance.

EpiMatrix, a T-cell epitope mapping algorithm, is based on matrices of coefficients that correspond to the preferred amino acid residues for each HLA allele. The tool has been successfully applied to the analysis of previously published epitopes [21], and in the prospective selection of epitopes from HIV [22], Mycobacterium tuberculosis [23], Tularemia [24] vaccinia virus [25], Burkholderia ([26], http://immunome-research.net/) and H. pylori [27]. EpiMatrix also been compared in a head-to-head study with other online tools and was found to be more accurate for a large database of well-defined class I and class II T cell epitopes [28]. In the current project, we performed a systematic review of epitope distribution in the entire EV71 polyprotein, using EpiMatrix and ClustiMer (a related algorithm for finding promiscuous T cell epitopes) to analyze our EV71 isolate (FY573, HM064456). Thirty-seven epitope clusters were predicted and subsequently examined for immunogenicity by performing ELISpot assays using PBMCs from healthy adults. Fifteen epitopes elicited detectable T cell responses in these donors, and sequence analysis revealed two dominant epitopes that were located in the capsid region. This epitope dominance correlates with the high degree of sequence conservation among the enteroviruses, suggesting that poliovirus vaccination in early childhood or infection with other enteroviruses may determine the immunodominance of CD4+ T cell responses to EV71 infection and to vaccination with HFMD vaccines.

**Figure 1:**
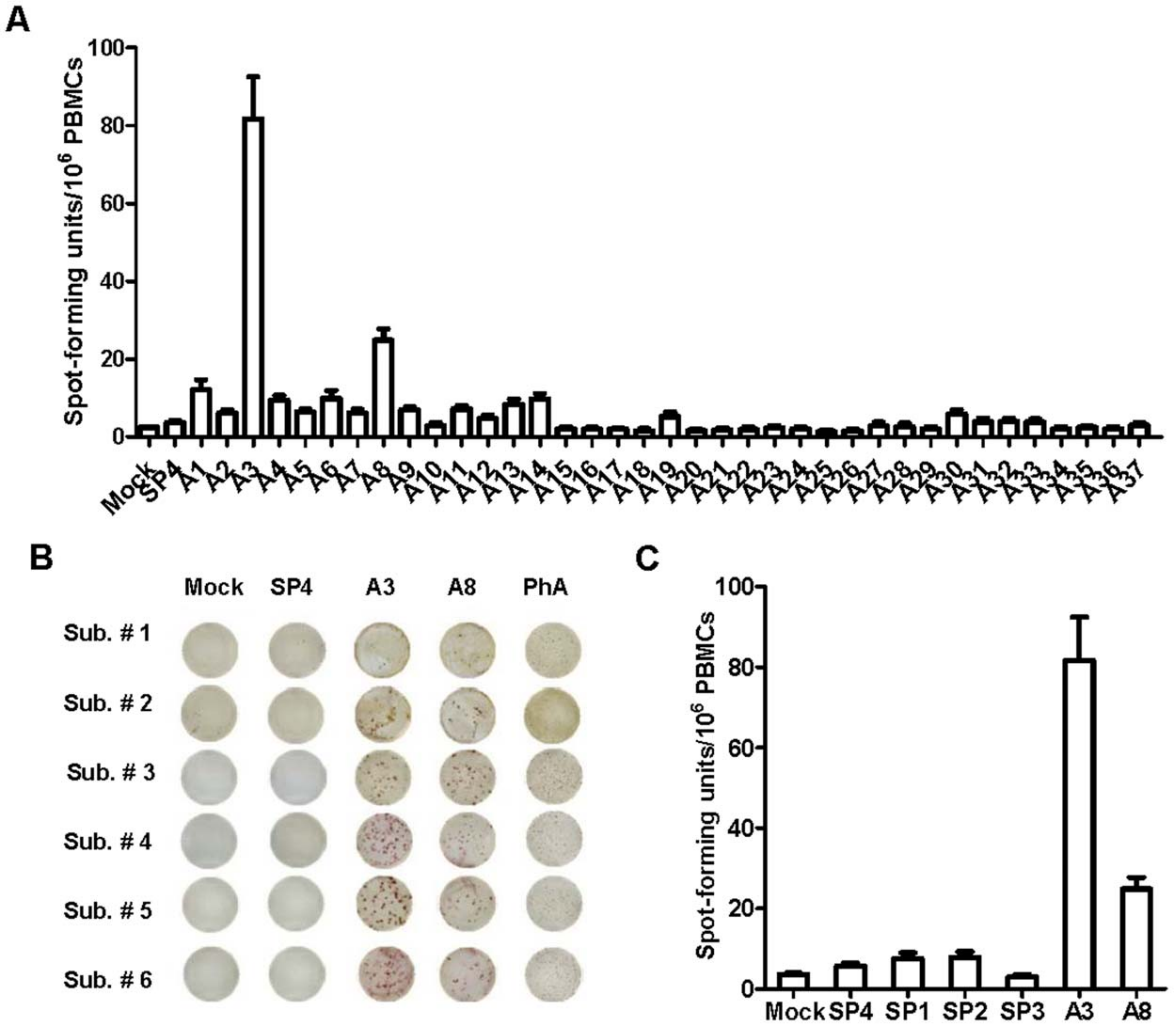
Identification of two dominant EV71 CD4+ T cell epitopes. (A) The average of spot forming units of PBMCs from 6 adult donors stimulated with 37 predicted EV71 CD4+ T cell epitopes and a scrambled peptide SP4. (B) These photos of ELISpot wells are examples of the responses to A3, A8, SP4 and PHA. (C) On average, the spot forming units of A3 and A8 were significantly higher than previously identified CD4+ T cell epitopes, SP1, SP2 and SP3.

## Materials and Methods

### 1. Human Subjects and PBMCs

PBMCs from anonymous adult donors were purchased from the Shanghai Red Cross Center. PBMCs were also separated from blood samples of HFMD patients at hospital admission and were collected in the Children's Hospital of Fudan University. All the HFMD patients were positive for EV71 in a RT-PCR assay. Written informed consent was obtained for the use of serum samples from all participants (or their parents) involved in this study. This study was approved by the ethics committee of the Children's Hospital of Fudan University in accordance with local governmental guidelines and institutional policies.

**Table 1:** Characteristics of immunogenic peptides.

Peptide	Sequence	Cluster Address	Location	% Reactive Donors	SI* Average ± SD
A1	NAQFHYLYRSGFCIHVQ	164–180	VP2	50	2.8±3.3
A2	ADGFELQHPYVLDAGISISQL	224–244	VP2	16.7	1.1±1.0
A3	PHQWINLRTNNCATII	248–263	VP2	**100**	**29.8±18.2**
A4	HCNFGLLVVPISPLD	278–292	VP2	50	1.8±1.5
A6	TGSFMATGKMLIAYTPPGGPLP	445–466	VP3	16.7	2.5±3.4
A7	IWDFGLQSSVTLVIPWISNTH	479–499	VP3	33.3	1.1±1.2
A8	NTAYIIALAAAQKNFTMKL	533–551	VP3	**83.3**	**7.3±3.7**
A9	SLAWQTATNPSVFVK	733–747	VP1	33.3	1.5±0.9
A11	NQNYLFKANPNYAGNSI	833–849	VP1	16.7	1.5±1.4
A12	SRDLLVSSTTAQGCDT	899–914	VP1	16.7	1.1±1.0
A13	HYPVSFSKPSLIYVE	933–947	VP1	50	1.9±1.3
A14	SREVEALKNYFIGSE	1034–1048	2A	**66.7**	**2.8±1.1**
A19	GLEWVSNKISKFIDWL	1128–1143	2B	33.3	1.1±1.7
A30	DSVYLRMAFGHLYETF	1914–1929	3D	50	1.4±1.6
A31	GHLYETFHANPGTITGSA	1923–1940	3D	16.7	0.2±1.1

* SI represents the fold increase in number of spot-forming cells over background.

### 2. Synthetic Peptides and Sequence Analysis

Peptides representing the 37 epitopes predicted by EpiMatrix, variants of A3 epitope, along with those previously indentified (SP1, SP2, and SP3) and a scrambled control (SP4) [19], were purchased from the HDbiology company (Shanghai, China). All the peptides were >95% pure as assessed by high-performance liquid chromatography. The peptides were firstly dissolved in dimethyl sulfoxide (DMSO, Sigma, USA) to provide as stock solution and then diluted in PBS to obtain a 4 µg/ml working concentration. To identify similar epitopes from other enteroviruses, the amino acid sequences of the synthetic peptides were used as query sequences in a protein-protein BLAST (blastp 2.2.17) search using the National Center for Biotechnology Information database. Sequence information of viral isolates used in this study can be queried in UniProt Knowledgebase. EV71 isolates include: ADG57604.1, ADG57603.1, ACF21980.1, ACD63041.1, ADC84176.1, ADC84177.1, ACI25378.1, ADD84781.1, ADX87405.1, ACM47545.1, ADC54995.1, ACF60581.1, ADG57604.1, ADG57603.1, AAR32993.1, ACU45380.1, ACU45379.1, and Q66478. The other enterovirus isolates included: CVA4 (Q66478), CVA9 (P21404), CVA16 (ABX55895.1), CVB3 (P03313), CVB5 (Q03053), CVB6 (Q9QL88), EV5 (Q9YLJ1), EV15 (Q6W9G3), EV30 (Q306E5), Poliovirus 1 strain Sabin (CAA24465.1), Poliovirus 2 strain Sabin (X00595.1), and Poliovirus 3 strain Sabin (Q84792).

**Figure 2:**
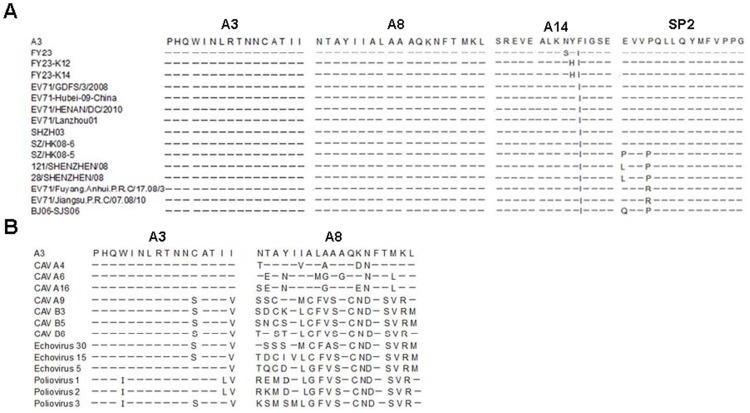
The dominant A3 epitope is highly conserved among human enteroviruses. (A) The A3 and A8 epitopes are exactly conserved across EV71 isolates, other HFMD-related viruses, and polioviruses. A14 was not as well conserved. Of the EV71 strains shown, all except for the EV71 prototype BrCr strain were isolated in the recent HFMD outbreaks. (B) A3, but not A8, was conserved among the other enteroviruses.

**Figure 3:**
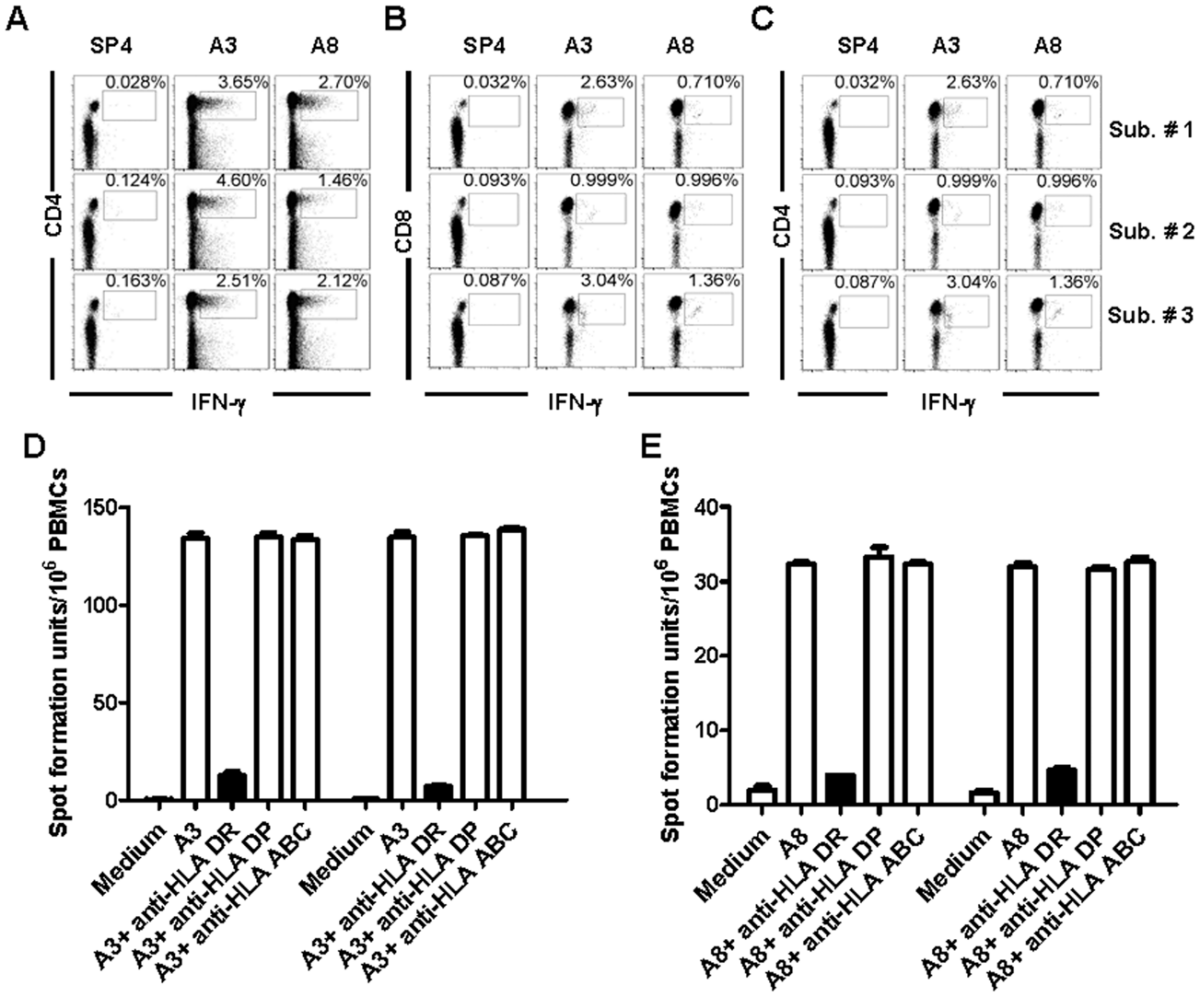
Both A3- and A8-specific T cells are HLA-DR-restricted CD4+ T cells. (A) and (B) PBMCs from adult donors were stimulated with A3 or A8 peptides and then expanded with recombinant IL-2. (A) The stimulated PBMCs were stained with fluorescently labeled antibodies against CD3, CD4, CD8 and IFNγ before flow cytometric analysis. Antigen-specific IFNγ production was primarily associated with CD4+ T cells. (B) CD8+ T cells produced negligible level of IFNγ in response to A3 or A8. (C) A3 and A8 can induce strong CD4+ cell responses in HFMD patients infected by EV71. (D) and (E) ELISpot assays were performed to examine IFNγ production by PBMCs which were stimulated with A3 (D) or A8 (E) peptides alone or in presence of blocking antibodies against HLA-DR, HLA-DP, or HLA-A, B and –C (HLA-ABC). The results were shown as average spot forming units ± standard deviation (n = 3). Only anti-HLADR antibodies abrogated the ELISpot responses, demonstrating that the responses to EV71 are Class II-restricted.

### 3. IFN-γ ELISpot Assays

ELISpot assays were carried out *ex vivo* with PBMCs as described previously by others [29]. Briefly, PBMCs were cultured with the individual predicted peptides or the scrambled peptide SP4. For validation of the newly predicted CD4+ T cell epitopes, 1.5×10^6^ PBMCs were plated per well in triplicate and incubated overnight at 37°C, 5% CO_2_, with each peptide at 4 µg/ml. The wells containing PBMCs with DMSO or 20 µg/ml PHA (Sigma) served as negative or positive controls, respectively. Note that only 3×10^3^ PBMCs were used in the PHA culture. The spot-forming units (SFU) of the plates were read on an automated ELISpot reader (Cellular Technology Limited), and the results were presented as spot-forming units (SFU) per 10^6^ PBMCs. To examine HLA restriction, 5 µg/ml antibodies against HLA-DP (Abcam), HLA-DR (Biolegend) and HLA-A, -B and -C (Biolegend) were added during the peptide stimulation.

**Figure 4:**
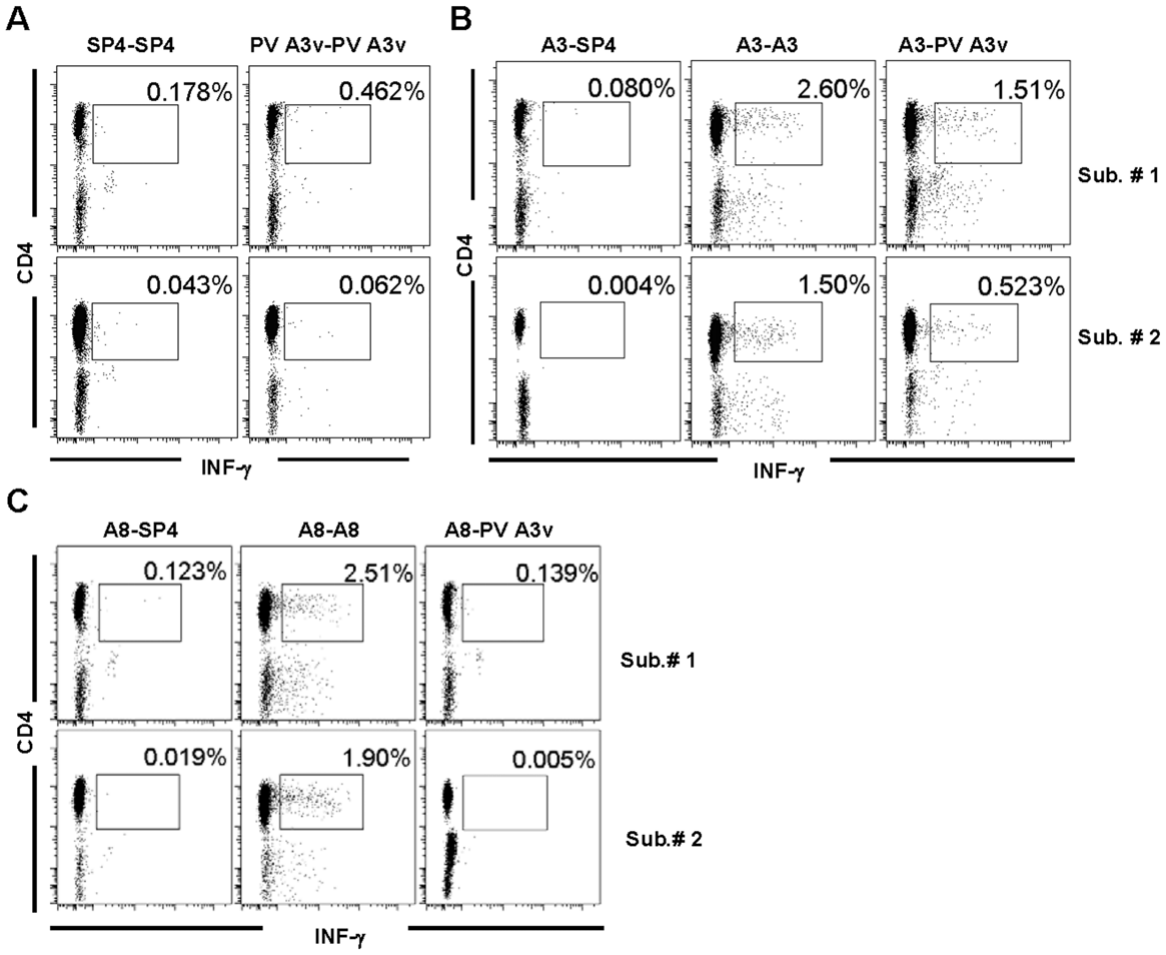
EV71 A3 or A8-specific CD4+ T cells respond to the poliovirus 3 Sabin (PV3) homolog, A3v peptide. PBMCs from two adult subjects were cultured in the presence of EV71 A3, EV71 A8, PV3 A3v, or SP4 peptides for 4 days and then with recombinant human IL-2 for additional 3 days. The cells were washed and re-stimulated with SP4, EV71 A3 or PV A3′, then surface-stained with fluorescent antibodies against CD3 and CD4 molecules and intracellularly for the production of IFNγ. Gated CD3+ cells are shown. (A) SP4 or PV3 A3v-stimulated cells were re-stimulated with the respective peptides. (B) EV71 A3-stimulated cells were re-stimulated with SP4, EV71 A3 or PV3 A3v, respectively. (C) EV71 A8-stimulated cells were re-stimulated with SP4, EV71 A8 or PV3 A3v, respectively. The above results were representative data from three independent experiments.

### 4. Intracellular Cytokine Staining and Flow Cytometry Analysis

Two million PBMCs were cultured with 4 µg/ml each peptide for 4 days. The cells were then cultured with 50 units/ml recombinant human IL-2 (R&D Systems) for an additional 3 days. PBMCs from the same patients, which were cultured with SP4 or with 10 ng/ml PMA (Sigma) and 500 ng/ml ionomycin (Sigma), served as negative and positive controls respectively. The cells were washed and re-stimulated with 4 µg/ml indicated peptides in the presence of 2 µg/ml brefeldin A (Sigma) at 37°C, 5% CO_2_, for 6 hours. Cells were stained with fluourochrome-conjugated antibodies against surface markers and then processed for intracellular cytokine staining (ICS) according to the manufacturer’s instruction (eBioscience) before being stained for IFN-γ. Fluorochrome-conjugated antibodies used were αCD4-APC, αCD8-PE-Cy7, αCD3-PerCP-Cy5.5, and αIFN-γ–FITC (eBioscience). All samples were processed on a BD LSR II and analyzed using FlowJo software.

To evaluate the cross-reactivity between A3 epitopes of EV71 and poliovirus, PBMCs that were cultured with EV71 A3 peptide and IL-2 were re-stimulated with 4 µg/ml EV71 A3 or A3 variant (A3v) of polioviruses in the presence of 2 µg/ml brefeldin A (Sigma) at 37°C, 5% CO_2_, for 6 hours. Re-stimulation with 4 µg/ml SP4 or with 10 ng/ml PMA (Sigma) and 500 ng/ml ionomycin (Sigma) served as negative and positive controls respectively. The re-stimulated cells were then processed for surface and intracellular staining.

### 5. Statistical Analysis

Proportional data were analyzed using Pearson's chi-squared test. Student’s t-test was used to determine statistical significance. All analyses were performed using SPSS software (version 11.5; SPSS). A difference with p<0.05 was considered to be significant.

## Results

### A3, A8 and A14 are Dominant Epitopes of EV71 Polyproteins

The entire primary amino acid sequence of an EV71 strain (FY573) that was isolated from a patient in Fuyang city in 2009 by our lab was subjected to analysis by the EpiMatrix and ClustiMer algorithms [30]. Thirty-seven promiscuous EV71 CD4+ T cell epitopes were predicted, of which 11 were found in structural viral protein regions, including VP1, VP2 and VP3 regions. The remaining predicted epitopes were located throughout the 2A, 2B and 3D proteins.

Human adults are about 80% seropositive for EV71, suggesting previous exposure [2]. PBMCs from 6 healthy anonymous (blood bank) adult donors were obtained and assayed by ELISpot for IFN-γ response to the individual predicted epitopes. Responses to the three previously published epitopes, SP1, SP2 and SP3, were examined in parallel. SP4 served as a scrambled control. In all, each of the 15 predicted epitopes elicited IFN-γ production by PBMCs from at least one of the donors (stimulation index (SI) ≥ 2.0) (Table 1); this level of response is slightly lower than, but consistent with, previous levels of response to EpiMatrix- and ClustiMer-predicted epitopes from viral pathogens [36]. Over two thirds of the immune responses (11/15) were to epitopes located in capsid protein region. Among these, epitopes A3, A8 and A14 elicited much higher immune responses as measured by SI and SFUs than the other epitopes (Figure 1A and 1B). All 6 adult donor PBMCs responded well to A3, with a maximum SI of 52.4 and a maximum of 214 SFU/10^6^ PBMCs; A3 was therefore defined as the most dominant epitope. Responses to A8 and A14 epitopes were lower than to A3, however, their selected SI’s were as high as 10 and 4.3, respectively. To our surprise, donor responses to SP1, SP2 and SP3 were not significantly different from the mock culture and scrambled peptide, with ELISpot averages lower than 10 SFU/10^6^ PBMCs (Figure 1C). Only two subjects (33.3%) had a SI above 2.0 in response to SP1, while none of subjects responded to SP2 or SP3 epitopes.

### A3 and A8 Epitopes are Conservative among EV71 Isolates

Since EV71 is an RNA virus, it lacks a proofreading mechanism and thus rapid evolution of viral sequence has been observed in association with the outbreaks occurring across Asia in regular cycles, and virus gene subgroups seem to differ in clinical epidemiological properties [31]–[33]. Many individuals are exposed to enteroviruses during these outbreaks. Thus, we wondered whether the dominance of EV71 CD4+ T cell epitopes might be related to their sequence conservation among circulating EV71 isolates. To address this question, we aligned the amino acid sequences of SP2, A3, A8 and A14 epitopes in 15 EV71 strains, which were isolated during the HFMD outbreaks from 2001 to 2010, using the JanusMatrix tool. JanusMatrix searches for conserved T-cell receptor (TCR) facing sequences while allowing the HLA binding residues to vary, as long as the peptide is still presented by the same HLA [De Groot, Bailey-Kellog and Martin, unpublished data]. Both the A3 and A8 sequences maintained 100% conservation at the TCR facing residues in EV71 isolates, including the EV71 isolates that had been circulating in the recent HFMD outbreaks in China (Figure 2A). However, both SP2 and A14, which were less immunodominant epitopes, demonstrated more sequence variability between isolates.

The most common etiologic agents of HFMD are EV71 and CVA16. However, in many outbreaks, a number of additional enteroviruses, such as CVA4, CVA6 and certain echoviruses (EV), may co-circulate [4], [34]. Using the JanusMatrix tool, we evaluated whether co-circulating enteroviruses shared the same or significant homology within the amino acid sequences of A3, A8 or A14 epitopes. Detailed sequence analysis revealed that the amino acid sequence of A3 was identical in the VP2 proteins of CVA4, A6 and A16. In addition, the A3 sequences were also conserved in HFMD-unrelated enteroviruses, including CVA9, CVB3, CVB5, CVB6, EV5, EV15, EV30, PV1, PV2 and PV3; albeit with two to four amino acid differences that did not affect binding to HLA presentation of the epitope to the TCR. Thus, A3 is an identical CD4+ T cell epitope in EV71, CVA4, CVA6 and CVA16. In contrast, the A8 epitope was not conserved among these enteroviruses (Figure 2B). Altogether, the relative dominance of A3, as compared to A8, among these epitopes appeared to be related to its extensive conservation among enteroviruses.

### Both A3- and A8-responding T cells are HLA-DR-restricted CD4+ T cells

To further characterize the nature of the T cell response to A3 or A8 epitopes, we performed intracellular IFN-γ staining of PBMCs obtained from adults, which were stimulated *in vitro* with A3 or A8 and subsequently expanded with recombinant IL-2. Our flowcytometry analysis revealed that the frequency of IFN-γ-producing A3- or A8-specific T cells varied from individual to individual, with a maximum of 4.60% responding to A3 and 2.70% to A8. The majority of IFN-γ-producing A3- or A8-specific T cells from the three representative volunteers were CD4+ cells (Figure 3A). In contrast, CD8+ T cells produced only a negligible background level of IFN-γ in response to A3 or A8 in all the volunteers (Figure 3B). Thus, it appears that the A3- and A8-responding T cells are CD4+ T cells.

Next we evaluated the response of T cells from HFMD patients to A3 and A8 epitopes (Figure 3C). Consistent with our findings in healthy adult blood donor subjects, IFN-γ-producing cells of patients’ PBMCs in response to A3 and A8 epitopes were also CD4+ T cells. Note that the response levels varied in the examined three patients. In addition, the response levels of A3 and A8 epitopes had a tendency to correlate with each other.

We next investigated the HLA restriction of A3- and A8 epitope-specific CD4+ T cells. We cultured PBMCs obtained from adults with A3 or A8 peptides in the presence of blocking antibodies against HLA-DR, HLA-DP or HLA-A, B and -C, and performed ELISpot assays. Blocking with HLA-DR antibody inhibited IFN-γ secretion by PBMCs in response to A3 and A8 by 90.82% and 85% respectively. The inhibition effect was not observed when blocking antibodies against other HLA Class II molecules were used (anti-DP and DQ), or with irrelevant control anti-HLA-A, B and C antibody (Figure 3D). Thus, the A3 and A8 epitopes appear to be restricted by HLA-DR molecules.

### Cross-reactivity of EV71 A3-specific CD4+ T cells with A3v Epitope of Poliovirus

The EV71 A3 epitope differed from the A3 variant (A3v) epitopes of poliovirus (PV) Sabin strains 1, 2 and 3 by three amino acid mutations, respectively (Figure 2B). We evaluated the potential cross-reactivity between the EV71 and poliovirus epitopes by testing whether cells exposed to EV71 epitopes would respond to poliovirus (PV) epitopes. The A3v (variant) epitopes elicited little to no detectable IFN-γ production (Figure 4A). Nevertheless, PV3 A3v but not PV1/2 A3v elicited measurable levels of IFN-γ production by the PBMCs obtained from adults that were first stimulated with EV71 A3 epitope and expanded with recombinant IL-2 (Figure 4B). This result suggests that T cells responding to the EV71 A3 epitope cross-react with the PV3 A3v epitope, as was predicted by the JanusMatrix analysis. The cross-reactivity of the PV3 A3v epitope appeared to be specific to the EV71 A3 epitope, since the PV3 A3v epitope failed to elicit IFN-γ production of the PBMCs that were first stimulated with the EV71 A8 epitope (Figure 4C).

## Discussion

In this study, an immunoinforamtics approach was used to systematically predict CD4+ T cell epitopes in the entire polyprotein of a contemporary EV71 isolate. To our knowledge, this is the first time that immunoinformatics tools combining HLA class II prediction and promiscuity (EpiMatrix and ClustiMer) were used to evaluate EV71. Among the 37 predicted epitopes, 15 epitopes were found to elicit a T cell response in from HFMD-exposed adults. A3, A8 and A14 emerged as dominant epitopes in this analysis, and A3 was the most dominant epitope (recognized by all the tested donors). Epitopes A3 and A8 are located in the capsid regions of the VP2 and VP3 proteins, respectively. This epitope distribution is consistent with a previous study on other enteroviruses [20]. While protein abundance may be one reason for epitope dominance, we present evidence here that the dominance of these epitopes may be attributed to their relative conservation among circulating EV71 isolates, other HFMD-related enteroviruses, and poliovirus vaccine strains. Specifically, the A3 epitope is identical in HFMD-related enteroviruses, including CVA4, 6, and 16, and it has the propensity to bind to multiple HLA-DR alleles as predicted by EpiMatrix. It is interesting to note that, with the exception of EV71, all the HFMD-related enteroviruses cause only mild illness. Thus, it would be of interest to investigate whether there is an epidemiological and biological correlation between the primary exposure to mild HFMD-related enteroviruses and subsequence exposure to EV71, and whether the primary exposure mitigates the severity of HFMD, in future studies.

The A3 epitope is also highly conserved in the other HFMD-related and even HFMD-unrelated coxsackieviruses, echoviruses and polioviruses. Intracellular staining confirmed that the EV71 A3-specific CD4+ T cells also cross-reacted with the poliovirus A3v epitope. The A3v epitope is distinct from the previously identified poliovirus epitopes [35]–[37]. Nevertheless, the cross-reactive nature of these epitopes implies the potential for cross-reactive immune responses related to poliovirus vaccination or other enteroviruses in early childhood and immunomodulation of subsequent CD4+ T cell response to EV71 infection or vaccination.

Low T cell responses to previously identified SP1, SP2 and SP3 epitopes and to poliovirus A3 variants were unexpected. This result seems to contradict a previous study by Foo et al. However, their method, which utilized dendritic cells as antigen-presenting cells, may not be strictly comparable to our PBMC ELISpot assays. An additional explanation for the disparate results could be that our subjects express different HLA molecules, or that the frequency or affinity of memory T cells in the examined PBMCs specific to the previously published epitopes is lower than the epitopes that we identified. Nevertheless, the strong immune response of our subjects to the A3 epitope suggests that this epitope should be the focus of evaluation by more sensitive assays in future clinical studies.

While cross-strain immunity may not necessarily provide sterilizing immunity against new emerging infections, it may mitigate the severity of disease. For example, we demonstrated that cross-reactive influenza-specific memory CD4+ T cells were present prior to the introduction of pandemic H1N1, and have postulated that they play an important role in reducing the disease severity of pandemic H1N1 infection in humans [38], [39]. As shown here, poliovirus vaccination may have an impact on subsequent severity of HFMD disease [40]. Cross-reactivity between EV71 A3 epitope and the A3v epitope of poliovirus 3 Sabin strain, may lead to the stimulation of protective, cross-reactive T cell responses, limiting the severity of subsequent HFMD.
