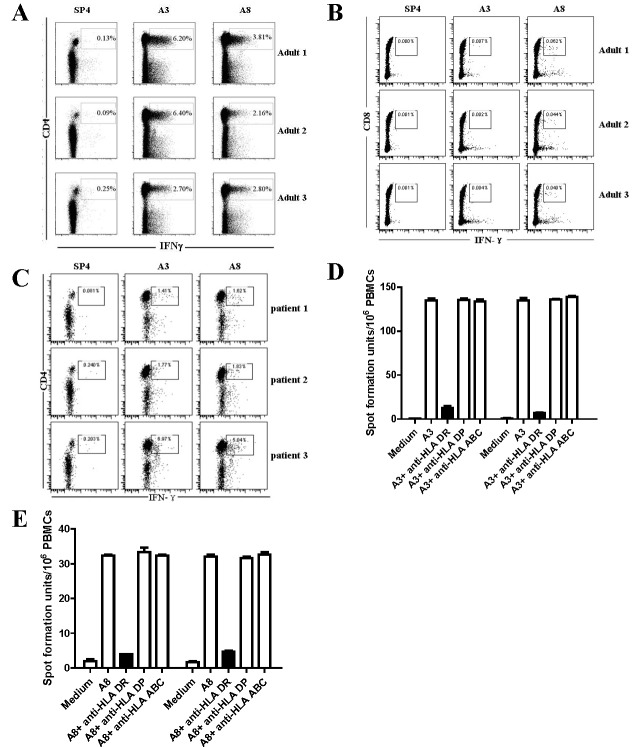# Correction: A Dominant EV71-Specific CD4+ T Cell Epitope Is Highly Conserved among Human Enteroviruses

**DOI:** 10.1371/annotation/434cdaa5-9654-4052-b325-fef0f31d4657

**Published:** 2014-01-13

**Authors:** Ruicheng Wei, Chunfu Yang, Mei Zeng, Frances Terry, Kai Zhu, Chunhui Yang, Ralf Altmeyer, William Martin, Anne S. De Groot, Qibin Leng

There are numerous errors in Figure 3. Please see the correct figure here: 

**Figure:**